# Fat-soluble vitamin intake from the consumption of food, fortified food and supplements: design and methods of the Belgian VITADEK study

**DOI:** 10.1186/s13690-017-0199-3

**Published:** 2017-05-16

**Authors:** Isabelle Moyersoen, Stefaan Demarest, Karin De Ridder, Jean Tafforeau, Carl Lachat, John Van Camp

**Affiliations:** 1 Department of Food Safety and Food Quality, Bruno Demeulenaer: Ghent University, Faculty of Bioscience engineering, Ghent, Belgium; 2 Scientific Institute of Public Health, Department of Public Health and Surveillance, Unit Surveys, Lifestyle and Chronic Diseases, Brussels, Belgium

**Keywords:** Online food frequency questionnaire, Fat-soluble vitamins, Dietary intake, Functional foods, Infants, Toddlers, Pregnant women, Lactating women

## Abstract

**Background:**

The adequacy of micronutrient intake is a public health concern, as both insufficient and excessive intake levels ﻿may result in adverse health effects. Data on dietary intake are needed to evaluate potential problems regarding inadequate intake at population level and to formulate effective public health and food safety recommendations. Assessing the intake of micronutrients in population subgroups such as infants, toddlers, pregnant and lactating women is challenging and requires specific approaches. This paper describes the Belgian VITADEK study, developed to assess fat-soluble vitamin intake from the consumption of food, fortified foods and supplements in four vulnerable groups namely infants, toddlers, pregnant and lactating women.

**Methods:**

Subjects were selected according to a multi-stage stratified sampling design with a selection of clusters proportionate to the population size. Recruitment occurred in collaboration with Belgian child health consultation centres and obstetric clinics. Participants were asked to complete a self-administered online food frequency questionnaire (FFQ) or to answer the questionnaire by phone if online participation was not possible.

The questionnaire was tailored to the specific diet of the different target populations. In order to capture vitamin intake from the consumption of foods, fortified foods and supplements, a market study was conducted to take an inventory of the fortified foods and supplements available on the Belgian market. The food list of the FFQ was based on both this inventory and the top 90% food groups that contribute to fat-soluble vitamin intake. Since fortification differs at brand level, food groups and subgroups were split up to the level of the brand of foods. Brand pictures were used as mnemonics to facilitate the recall of the consumed food items and portion pictures were used to facilitate the reproduction of the consumed portion sizes. Finally a composition table was compiled allowing for the computation of vitamin intake from all sources providing as such more accurate estimates of fat-soluble vitamin intake.

**Discussion:**

The results will allow assessing inadequate micronutrient intake by comparison of vitamin intake with dietary reference values. The data will further allow describing the most contributing food groups as well as the contribution of fortified foods and supplements to total vitamin intake. The data will enable evaluating whether infants, toddlers, pregnant and/or lactating women are reached by the actual Belgian fortification and supplementation programmes. Finally the retrieved data will reveal the potential for voluntary fortification and the need for future fortification and supplementation programmes.

**Electronic supplementary material:**

The online version of this article (doi:10.1186/s13690-017-0199-3) contains supplementary material, which is available to authorized users.

## Background

Deficiencies or suboptimal status of fat-soluble vitamins (vitamins A, D, E and K) are common in different population (sub)groups in Europe [1, 2]. Both insufficient and excessive intake levels can result in adverse health effects [3–7]. The reduction of the prevalence of micronutrient deficiencies, and as such fat-soluble vitamin deficiencies, is part of food and nutrition policies in Europe as recommended in the European food and nutrition plan 2015–2020 of the World Health Organisation (WHO) [8].

Due to a higher food consumption per kilogram body weight, infants and young children are exposed to higher levels of micronutrients. Infants and toddlers are therefore considered the most vulnerable by the European Food Safety Authority (EFSA). Similar concerns apply to pregnant and lactating women due to the effect of micronutrient intake on the unborn or breastfed child [9]. Micronutrients play an important role in growth and development. As a consequence infants, toddlers, pregnant and lactating women are more prone to adverse health effects due to inadequate vitamin intake. Deficiencies can cause intrauterine growth retardation, postnatal growth retardation, congenital malformations (vitamin A deficiency), rickets (vitamin D deficiency) and haemorrhage (vitamin K deficiency) [3–6].

These vitamin deficiencies can be avoided and should therefore not occur in Europe [2]. At a population level, fortification and supplementation programmes can be used as a strategy to overcome suboptimal intake levels. Yet, one has to be aware of the risk of excessive nutrient intake that may occur from the aggregated consumption of foods, fortified foods and supplements, and therefore apply such fortification and supplementation programmes with caution, whilst carefully monitoring their impact [10].

An effective supplementation or fortification policy should ideally be based on population intake data. These data can reveal target populations at risk for deficiencies or possible excessive intakes [7]. Such data may furthermore be used to evaluate the effect of fortification and supplementation programmes. The different target populations should be reachable by the actual programs, while non-effective programmes are a waste of resources possibly leading to excessive intakes [10].

Assessing the intake of a specific nutrient demands that all relevant sources of the specific nutrient are taken into account i.e. foods, fortified foods and supplements [11, 12]. Information is therefore needed on the actual supply of fortified foods and supplements on the market. Since fortification may differ at the level of the brand of a certain product, brand information is required. To enable computation of the intake of a specific nutrient from food, fortified foods and supplements, a composition table needs to be compiled with the exact amount of that nutrient in each of the foods and supplements under consideration [12]. Since the market of functional foods is a rapidly evolving market, the supply of those products as well as their fortified nutrient content is subject to permanent modification [13]. A functional food database should therefore be composed as close as possible to the moment the survey is carried out [11, 12].

Collecting food consumption data in specific target populations requires a specific methodology. Reaching those target populations might be challenging and often demands a distinct recruitment procedure through appropriate channels. Furthermore, the dietary assessment method should account for the specific diets (menu and portion sizes) of the target populations. Finally, some fortified foods are designed especially for certain target populations and should therefore be listed separately. It may therefore be more efficient to conduct an ad hoc survey in each of the four target populations.

Currently, no data are available in Belgium on the intake of fat-soluble vitamins from food, fortified food and supplements. The VITADEK study tries to fill the void. It has been designed to assess the intake of fat-soluble vitamins (A, D, E and K) from the consumption of food, fortified food and supplements in four Belgian populations subgroups i.e. infants, toddlers, pregnant and lactating women.

Reporting of dietary assessment and nutritional epidemiology findings often lacks detail in the description of the applied method which hampers the understanding of the results. Recent guidelines recommend adding sufficient detail of the methods used to select participants and assess dietary intake to facilitate the interpretation of the findings and enable replication of studies [14].

This paper describes the methodological choices made in composing the sample and the dietary assessment method as well as the preparatory work needed prior to the dietary assessment e.g. market research, construction of the questionnaire and compilation of the attached food and supplement composition table. This paper further reflects on the implications of the methodological choices, on the sample size and their consequence for the results of the study.

## Methods

### Target population

The target population of the survey is comprised of infants (0–12 months), toddlers (13–36 months), pregnant and lactating women residing in Belgium. Data on infants and toddlers were collected through their proxy, e.g. one of the parents or caretakers.

### Sampling frame

Belgian Child health consultation centres (CHCCs) were used as the sampling frame to select infants, toddlers and lactating women (lactating women were retrieved through their new-born).

Child health consultation centres offer free services of health consultation and vaccination programmes for each child born or residing in Belgium. CHCCs are organised at the municipality level and have a high coverage. About 86% of children 0–12 months old, 82% of children 13–24 months old and 63% of children 25–36 months old visit these centres on a regular basis [15]. Although infants and toddlers could have been selected through the national population register (NPR), this method has not been chosen, because the CHCCs provided the opportunity not only to select the infants and toddlers, but also to identify lactating women. Furthermore﻿ it has been suggested that a personal contact may increase the participation rate [9].

Obstetric clinics were used as sample frames for pregnant women due to a high coverage rate and the lack of existing registration lists for pregnant women. In Belgium a clinical consultation is advised at 12, 20, 30 and 34 weeks of pregnancy.

Choosing CHCCs and obstetric clinics as sampling frames implies that children or women not visiting these centres or clinics were excluded from the survey. Due to logistical and financial constraints, the German-speaking Community of the Belgian population, representing 0.5% of the yearly births in Belgium only, could not be included in the survey.

The questionnaire was available in the main Belgian languages i.e. French and Dutch. People that did not master any of those languages were excluded from the survey.

### Sample size

Determination of insufficient or excessive intake levels of vitamins involves an assessment of dietary intake data against dietary reference values i.e. the estimated average requirement (EAR) and the upper intake level (UI). This implies an evaluation at the tails of the intake distribution and enquires as such a precise estimation of the upper and lower percentiles.

The accuracy of estimates of high or low dietary intake levels (percentiles 2.5^th^, 5^th^, 95^th^ and 97.5^th^) depends on the sample size. The minimum sample size for the 95^th^, 97.5^th^ and 99^th^ percentile, with a statistical significant level of 1% was estimated as 130, 263 and 662 respectively [9]. For the lower percentiles, the same assumptions can be used. In order to harmonise the food consumption data collection in Europe, the guidance documents of EFSA advise to select at least 130 participants in each sex-age class and in population subgroups such as pregnant and lactating women [9].

In order to improve statistical power, we aimed for 300 subjects in each target population divided over 30 CHCCs/hospitals, meaning that we needed to select 10 subjects per CHCC/hospital per target population.

Participation in the VITADEK study is voluntary. In European dietary surveys, participation rates vary from 28–98% and have been decreasing recently [9], therefore a tenfold of the targeted number of subjects (10 per CHCC/hospital per target population) was aimed for.

### Sampling design

The sampling design for infants, toddlers and lactating women was distinct from that of pregnant women.

#### Infants, toddlers and lactating women

Infants, toddlers and lactating women were selected according to a multistage proportionate-to-size stratified sampling design [16]. First, a geographical stratification by province was executed, followed by a selection of municipalities and CHCCs within each province, and finally by the recruitment of 10 subjects per target group within each CHCC.

The country was first divided into 11 strata (10 Belgian provinces and the Brussels Capital Region) in order to ensure the representative geographical distribution of the sample. Within each province, municipalities were then ordered by population size (number of registered clients in the consultation centre(s) of the municipality) and selected using a systematic sampling approach. The number of CHCCs to be selected within each province was proportional to the number of births per province per year. The systematic sampling procedure guaranteed that both large and small municipalities and their corresponding CHCCs were included. While small communities only have one CHCC, larger communities might have 2 or 3 CHCCs. In the latter case, one CHCC was randomly selected. A total of 30 CHCCs were selected in the 11 strata. In order to recruit 10 participants per target group in each CHCC the first 100 clients visiting the centre were invited to participate in the study.

#### Pregnant women

In Belgium, obstetric clinics are organised in 103 hospital groups. In order to ensure a representative geographical distribution of the sample of pregnant women, the country was divided into the Flemish, the Walloon and the Brussels Capital regions. Hospital groups were again selected using a systematic sampling approach, with a selection chance of the hospitals proportionate to the population size of the region, i.e. number of births per region. Similar to the procedure used in infants, toddlers and lactating women, 30 hospitals were selected from each of which 10 participants were to be recruited.

### Recruitment

Doctors, social workers or collaborators of the child health consultation centres and gynaecologists and midwives at the hospitals played a crucial role in the recruitment of participants. Prior to the onset of the study, healthcare professionals of the selected CHCCs or hospitals received recruitment guidelines in order to ensure a standardised approach. During consultations they were asked to invite, per target group, the first 100 clients/patients or their proxies (in case of infants and toddlers) to participate in the study. Possible respondents received a brief explanation on the purpose of the study, an information brochure and a reply card. Respondents or their proxies were asked to fill out an online food frequency questionnaire. The questionnaire was accessible through a website mentioned in the brochure (https://vitamine.wiv-isp.be) or via a link sent to the respondent after entering their e-mail address on the reply card [17]. A completed card offered the possibility to send reminders by e-mail. A maximum of two reminders were sent to subjects that volunteered to participate but did not start to fill out the questionnaire or did not complete the questionnaire. The time interval between correspondence was 1–2 weeks. Reply cards also offered the possibility to the respondents to denote their wish to answer the questionnaire by phone. As such, computer or internet illiterate respondents were not excluded from the survey.

The recruitment procedure of infants, toddlers and lactating women differed slightly in the Flemish Region: a letter with brochure and reply card was sent to the subjects, 1 or 2 weeks prior to their visit of the CHCC. During this visit, clients were then reminded to participate in the study.

A first recruitment wave took place between October and December 2015. Because of some logistic constraints and the fact that contact details were missing for the majority of the invited subjects, the obtained sample was not sufficient. A second recruitment wave (with 100 brochures per CHCC) was therefore organised in each of the selected CHCCs between February and June 2016.

The selection of pregnant women took place between November 2015 and February 2016. A second wave with new hospitals was organised from September till December 2016.

### Study design

The VITADEK-study is a cross-sectional study. An online FFQ was developed to assess fat-soluble vitamin intake from the consumption of food, fortified food and supplements.

For the purpose of this survey a recall period of one month was chosen since dietary habits rapidly change in early life years and the periods of lactation and pregnancy are limited in time. However, consumption of supplements and rarely eaten foods, with high contribution of one of the vitamins under study (liver, weight-loss products, energy drinks (type Redbull,..), were enquired with a recall period of one year.

Due to different eating patterns (menu and portion sizes), different questionnaires were developed for the three target populations, i.e. infants, toddlers and adult women (pregnant and lactating women combined) [18, 19]. The infants’ questionnaire consisted of fruits, vegetables, meat and fish, suitable for children of this age and typical infant food like baby cereals, infant milk and follow-on milk. Likewise the toddlers’ questionnaire contained typical food items like toddler’s biscuits and cereals. Finally, the adults’ FFQ differed from the toddlers’ FFQ in that it contained typical foods such as weight loss products and energy drinks as well as larger portion sizes.

The online questionnaire was designed such that it was also suitable for the computer-assisted telephone interview without adaptation of the questions. In order to ensure a similar assessment method, respondents who chose to answer the questionnaire by telephone received a photobook containing the same pictures as the ones presented in the online questionnaire. Subsequently, they were contacted by dieticians and were administered the same questions as for the online version.

The study obtained approval from the Ethics committee of Ghent University hospital (reference B670201525592). Participants had to mark their informed consent when starting the online questionnaire. For the computer-assisted telephone interviews, a signed consent was obtained from the participants.

### Development of the questionnaire

For the aim of this study, the questionnaire had to be capable of assessing fat-soluble vitamin intake from the consumption of food, fortified foods and supplements [11]. To obtain this goal, the food list of the FFQ was compiled using a data-based approach combined with the results of a market research [18].

#### Market research of voluntarily fortified foods and supplements

A comprehensive market research was conducted to list all foods fortified with fat-soluble vitamins by investigating their ingredient list for addition of vitamin A, β-carotene, vitamin D, E and/or K. Fortification often differs per brand or variety of a product. Therefore the inventory had to be done accurately. Within a certain food group (for instance cereals) each brand and each variety within those brands had to be strictly checked for the addition of fat-soluble vitamins.

Food items from the five most important supermarket companies in Belgium were listed. Through a label study, fortified food items were detected and a food composition table was compiled with values for added nutrients. The market research revealed fortification with fat-soluble vitamins in margarines and spreadable fats, infant milk and follow-on milk, dairy products and substitutes, breakfast cereals, biscuits, fruit juices, lemonades, sports and energy drinks and weight loss products.

To screen the actual supply of food supplements, the supplement supply of drugstores and supermarkets, as well as 15 pharmacists (5 in each of the three Belgian regions), were monitored. Supplements containing one or more of the fat-soluble vitamins and designed for the target populations were registered.

The market of fortified foods and supplements is a rapidly evolving market [13]. Between the market inventory and the startup of the study, a number of new foods fortified with fat-soluble vitamins were launched on the market while others disappeared. Therefore, the functional food inventory was created as close as possible to the assessment of food consumption, and regularly checked for new foods, brand name changes and new varieties.

#### Informative food list

No previous data were available in Belgium on the intake of fat-soluble vitamins. Therefore the Dutch national food consumption survey (DNFCS 2007–2010, data for women aged 19–69 years old) was used as database to identify food (sub)groups contributing 90% of total vitamin A, D and E intake. The DNFCS did not include data for vitamin K. The most important food sources for vitamin K were added to complete this list [20]. An additional file gives an overview of the food groups that contribute the most to vitamin A, D, E and K intake (see Additional file 1).

For each selected food subgroup the 90% most consumed food items and 90% most consumed brands were selected from the raw database of the Belgian national food consumption survey (BNFCS2014) (since the completed database was not yet available) [21]. This list was complemented with all the fortified food brands resulting from the market inventory if not already included in the questionnaire.

The list of dietary supplements contained all types of supplements with fat-soluble vitamins i.e. single vitamins (vitamins A, D, E and K), vitamin AD, vitamin KD, omega 3 supplements, Ca-supplements, multivitamins and mineral vitamin complexes. Per type of supplement a list of brands, resulting from the market research, was included in the questionnaire. This list was confined to supplements designed for the specific target groups under study.

The list of supplements and fortified foods might not be complete. For this reason the questionnaire provided open-ended questions per food subgroup allowing the respondent to add a fortified food or supplement brand not mentioned in the list.

#### Compilation of the questionnaire

Fat-soluble vitamin intake was assessed using an online food frequency questionnaire developed in Lime Survey version 2.06 + Build 150825. The questionnaires were available in French and Dutch.

Each questionnaire consisted of three parts:

- a general part to obtain sociodemographic characteristics of the respondents;
- the food frequency part of the questionnaire enquiring the frequency of consumption and portion size of a finite list of food items.
- a food supplement part assessing the consumption of food supplements

#### General part of the questionnaire

In the general part of the questionnaire questions related to the socio-demographic characteristics of the respondents (age, country of birth, educational degree of respondent and partner and the municipality of the CHCC or obstetric clinic they consulted) were asked. Some additional target specific questions were asked, i.e; the childcarer during the daytime, duration of the pregnancy and the age of the newborn child (in case of lactating women). Finally, the general part enquired whether or not subjects had been reading the ingredient lists of the products they consumed as well as their reason for buying fortified foods. Multiple answer choices to the latter question were: “because I like the product”; “since extra vitamins are added and I think they are good for my health”; “because extra vitamins are added and I think I can use some supplementation of vitamins”; “other reason”.

#### Food frequency part of the questionnaire

The food frequency part of the questionnaire consisted of a defined list of food items ordered in food groups and subgroups, for which the respondents were asked to recall their frequency of consumption and consumed portion size over the past month.

Advanced branching ensured that the respondent was only asked relevant questions. If a respondent did not consume foods of a certain food group (for instance meat) in the past month, no further questions on this food group were presented. When respondents had eaten foods from a certain food group, they could select one or more food items or brands eaten. Brand or variety pictures were shown as a mnemonic to facilitate precise identification of the consumed brand or variety [11].

Consumption frequency and portion sizes were asked for the selected foods only. Frequency of consumption included the following categories: every day, 5–6 times per week, 3–4 times per week, 1–2 times per week, 3–4 times per month and 1–2 times per month. Food items that are rarely eaten, but with substantial contribution to fat-soluble vitamin intake (ex. liver, liver products, weight loss products) were inquired over a period of one year. This implies the addition of frequency categories like: 5–6 times a year, 3–4 times a year and 1–2 times a year.

Portion sizes were identified as kitchen measurements (spoon, glass,..), units (apple, brick, can,..) or portion pictures. These pictures were extracted from the GloboDiet® picture book and the Pancake study picture book (validated for children of 0–10 years old) used in the BNFCS2014. GloboDiet® is a computerised 24 h recall programme to collect standardised individual consumption data in Europe [21].

Some food items, such as milk, are typically consumed at different daily occasions but in different portion sizes, e.g. a glass of milk as a drink, a cup of milk added to breakfast cereals or a dash of milk in coffee or tea. As a consequence, portion sizes of such food items are difficult to reproduce. The questionnaire accounts for these different consumption occasions and provides a different question per consumption occasion for these food items.

Concerning cooking fats, only the kind of fat used for preparation (i.e. butter, margarine or oil) was enquired as well as the type of butter (whole cream, semi-skimmed) and/or type of oil (olive oil, sunflower oil,…). Since FFQs are potentially prone to substantial measurement error when it comes to the consumption of cooking fat, frequency of consumption and portion sizes of cooking fat were not enquired. Fat uptake of the consumed food items that were mostly eaten prepared, was calculated based on the weight yield factors identified by Bognar et al. [22]. Yield factors were used to account for the percentage of weight change in foods due to cooking.

Each food subgroup ended with an open-ended question where the respondent could indicate other foods from the same food group eaten in the past month.

The whole questionnaire took on average 25 min to complete for the infants’ questionnaire and 50 min for the adults’ and toddlers’ questionnaires.

A schematic representation of the design of the food frequency questionnaire is presented in an additional file (see Additional file 2).

#### Supplement part of the questionnaire

In this part of the questionnaire, respondents were asked about their consumption of food supplements during the past year. Respondents could select the type(s) of consumed supplements from a generic list of supplements (vitamins A, D, E, K, AD, DK, multivitamins, vitamin and mineral complexes, Ca-supplements and omega 3 supplements). Per selected type of supplement a list of brands appeared, illustrated with pictures, to facilitate recognition of the consumed brand. Period of consumption (spring, summer, autumn, winter, during the whole year), administration form (effervescent tablet, drops, tablets, syrup,…), amount per day and duration of consumption (in days, weeks, months or other) were enquired as well. Finally the reason of consumption was asked by means of a list of predefined answers.

### Compilation of the food composition table

**Fig. 1:**
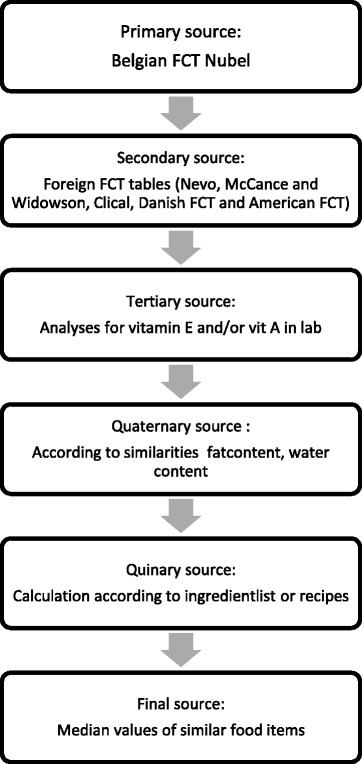
Successive steps in the compilation of the food composition table, VITADEK study, 2015–2016

In order to compute fat-soluble vitamin intake, a food composition table (FCT) needed to be compiled. For each food item of the FFQ, values were retrieved for vitamin A, β-carotene, vitamin D, E and K. A protocol was developed to ensure that a uniform method was applied. Figure 1 presents an overview of the successive steps followed in the compilation of the food composition table. The Belgian food composition table Nubel was used as basis [23]. Since corresponding food codes in the table often lacked data on fat-soluble vitamins, five other databases were used in the following order: 1) NEVO, the Dutch food composition database, 2) McCance and Widdowson’s composition of foods integrated dataset, the UK food composition table, 3) Clical, the French composition table, 4) the Danish food composition table, and 5) the American food composition table [24–28]. When data were missing for the first source, the next source was consulted until the appropriate values were found.

All nutritive values were derived from the tables except for the values of the fortified nutrients. Those values were taken from the ingredient list of the food item. Fortified values were marked so that contribution of fortified foods in total vitamin intake could be derived afterwards.

For all prepared foods such as meat, meat substitutes, eggs, fish and vegetables, cooking fats were not taken into consideration when compiling the FCT. Fat uptake was calculated afterwards following Bognar et al. [22]. Vitamin values were thus obtained from food items that were prepared fat-free. This implies the use of preparation methods such as boiling, preparation in the microwave and in the steamer. Because the FFQ does not take the preparation or cooking method of foods into account, the latter was determined using a data-based approach. Data of the BFCS 2014 specifies the number of people that consumed a certain food item raw or prepared (baked, steamed, cooked etc.). Depending on the method that was most frequently used, the vitamin values of either the prepared food item or the raw food item were included in the FCT. When only the raw food item was available in the composition table, this was marked. Thus, the nutrient composition of the cooked food could be calculated later on from the raw food by applying the nutrient retention factor (calculated by the True retention method (%TR)). The nutrient retention factor accounts for the additional loss of vitamins due to the cooking method [29].

After consulting these six composition tables (Nubel and five international tables) a substantial number of food items still contained some missing values. The products with missing values for vitamin A (retinol and carotenoids) and E were analysed in the nutriFOODchem lab of Ghent University, while missing values for vitamin D and vitamin K were derived from ingredient or recipe-based calculations, through similarities or as a median value.

Using the method of Bolton-Smith et al., missing values for vitamin K and D were calculated based on the vitamin K or D values of their components [20]. The ingredient list was consulted in order to extract the proportion of each of the main components. If no such list was available, a cookbook was consulted to derive the proportion of ingredients. Ingredient-based calculations were applied for vitamin K and vitamin D values in cookies and spreads and for vitamin K values in sauces, bread, pancakes, waffles, pastry and some milk substitutes. In this process, the following assumptions of Bolton-Smith were applied. When the type of oil or margarine was not stated or different oils/margarines were used depending on the brand of the food, blended vegetable oil or margarine was chosen. The contribution of spices, dried herbs and seasonings were assumed negligible [20].

Missing values for vitamin K in some cheese and fish were derived from products with similar fat and/or water content [20, 28]. Finally, lacking values for foods that were part of a group of similar food items (for example cereals), were determined based on median values. This was applied for vitamin K values in cereals.

## Discussion

This paper describes the methodology of the VITADEK study designed to assess fat-soluble vitamin intake in 4 vulnerable groups of the Belgian population, i.e. infants, toddlers, pregnant and lactating women. To our knowledge, data on intake of fat-soluble vitamins in those target populations is scarce, especially data taking into account all sources of these micronutrients, i.e. foods, fortified foods and supplements.

Methodologically it is a challenge to conduct dietary surveys in infants, toddlers, pregnant and lactating women. Reaching these target populations demands a distinct recruitment procedure while assessing their dietary intake demands for age-appropriate measurement instruments. These target populations are therefore often omitted in national consumption surveys and require the implementation of ad hoc surveys [9].

Even though infants, toddlers, pregnant and lactating women are difficult to reach, the VITADEK study was able to reach enough participants in each of the target populations to comply with the EFSA sample size guidelines (*N* = 130) [9]. In total 508 infants, 223 toddlers, 141 pregnant women and 132 lactating women were included in the study.

The VITADEK study was designed to allow for a representative sample of the participating CHCCs and hospitals by means of a multistage proportionate-to-size sampling design. The stratified sampling procedure ensures a geographical representation of the country while the systematic sampling ensures the inclusion of both small and large CHCCs/hospitals in the sample.

### Strengths

A particular strength of the VITADEK study is that it made use of 3 different FFQs tailored to the specific diet of each target population [18, 19]. We chose to develop online self-administered FFQs as a dietary measurement instrument because they are convenient to administer, budget-friendly and easy to adapt to the specific purpose of the study [30]. The questionnaires were also suitable for the computer assisted telephone interview without having to adapt the questions.

Another important strength of the VITADEK study is that it takes into account all food sources contributing to the total intake of fat-soluble vitamins. Fortified products and supplements may contribute highly to the intake of a nutrient even though they are only consumed by a smaller proportion of the population [11]. The food list of the FFQ was able to reflect the actual supply of fortified foods and supplements on the Belgian market. By means of a comprehensive market research prior to the onset of the study, and the the top 90% food groups that contribute to vitamin intake, the FFQ gave a detailed picture of the respondents’ diet considering key sources to fat-soluble vitamin intake. Furthermore, the use of brand and portion pictures added to the accuracy of the responses. Brand pictures allowed a precise identification of the consumed brands, while the use of validated portion pictures allowed for a more precise estimate of the consumed portion sizes. This added to the accuracy of the responses.

Finally, a composition table was compiled providing information on the most recent fat-soluble vitamin content of all supplements and fortified foods included in the questionnaires. Since the market of fortified foods and supplements is a rapidly evolving market, a comprehensive market study ensured the most recent nutritional label values were included in the table. Since national food composition tables often lack data on fat-soluble vitamins, a big effort was made to complete this table. This allows a more precise calculation of vitamin intake and thus provides better estimates of total fat-soluble vitamin intake [31].

### Limitations

Infants, toddlers and lactating women were recruited through CHCCs, while pregnant women were recruited through obstetric clinics. Although those sampling frames have a fair to high coverage, possible selection bias cannot be excluded. This will be evaluated during the analysis by comparison of socio-economic position parameter distributions in the acquired samples with the expected distributions in Belgium for the specific subpopulations.

Despite the great efforts that were made to select a representative sample, a large amount of subjects needed to be contacted in order to retrieve the required sample size. Subjects were invited to the study by medical caregivers of the CHCCs or the obstetric clinics. All subjects visiting the centres during recruitment period were invited to participate. Unfortunately, since recruitment was not in the hands of the researchers in charge of this study, it could not be controlled for. Furthermore, methodological constraints such as unreadable or incorrect e-mail addresses and some delays in returning the response cards, hampered the recruitment. Since it was not compulsory to fill out a reply card (people could also directly participate through the website mentioned in the brochures, without registering first by means of the reply card), people might also have forgotten to participate even when they intended to do so. Participants may have been more interested in the study and more concerned about their eating habits than non-participants. This might result in a bias and a probable overestimation of the average intakes. Possible confounding factors will be controlled for by investigating the population distribution for age and educational level.

Ideally the fieldwork should be organised in seasonal waves to capture inter-season variability [9]. A limitation of the study is that the fieldwork did not cover all seasons of the year. The recruitment procedure implemented the intervention of CHCCs and obstetric clinics, for whom too long a recruitment period would have posed too high a burden. This might result in a bias in the estimated average intakes and will be taken into account when discussing the results of the analysis.

Although a FFQ is generally regarded as the most cost-effective and practical instrument used to assess dietary intake, validation studies prove FFQs to be prone to substantial measurement error [32]. Due to the limited food list, lack of recipes and preparation methods, difficulties to recall the consumed portion sizes and the longer recall period, FFQs tend to over- or underestimate population dietary intake [19, 30, 33]. A combination of multiple 24 h recalls and a concise FFQ accounts for both the within-person variability, whilst offering the required level of detail [32]. The designed FFQ will not allow assessing fat-soluble vitamin intake as accurately as this combined method. Nevertheless, the developed questionnaire was adapted to overcome some of the general limitations of a FFQ. The food list was comprehensive enough allowing for a detailed image of the respondent’s diet. All sources of the vitamins under study were taken into account. Pictures were used as a mnemonic to accurately recognise the consumed fortified product or supplement. Finally, food items consumed in different portions on different daily occasions, such as milk, were questioned separately.

### Opportunities

The VITADEK study makes it possible to assess the adequacy of fat-soluble vitamin intake taking into account all possible sources of the vitamins under study. Furthermore, the retrieved data will bring forward some other valuable information. First, they will enable detecting the contribution of fortified foods and supplements to total vitamin intake. Then, they will reveal whether those who would have an insufficient intake when eating foods only (i.e. non fortified foods) are reached by the existing fortification and supplementation programmes [10]. Finally, they will make it possible to determine the options for future fortification and supplementation programmes [7].

Based on the results, policy recommendations can be formulated from a public health and food safety point of view. The estimated average requirements for fat-soluble vitamins are actually based on foreign studies. Dietary intake data are needed in order to (re)formulate supplement recommendations and to finetune the legislation on maximal permitted doses in supplements and fortified foods [34]. Furthermore, the data can be used to extend the Belgian Royal Decree (KB 2 Oktober, 1980) on the mandatory addition of vitamin A and D in spreadable fats and cooking fats. The current version of the decree only applies to margarines with fat content >80% and spreadable fats with fat content between 39 and 41%.

## Conclusion

This paper describes the methods of the VITADEK study in Belgium, 2015–2016. Data collected allow assessing fat-soluble vitamin intake from the consumption of foods, fortified foods and supplements. Due to the detailed food list and composition table, the VITADEK study will provide more accurate estimates of the population intake. Data will be evaluated against dietary reference values in order to detect inadequate intake levels in each of the population subgroups. Such data are needed in order to (re)formulate food safety recommendations e.g. regulations on safe addition of fat-soluble vitamins in functional foods and supplements. Data will also allow to (re)formulate public health recommendations e.g. mandatory fortification and supplementation programmes at the level of the target populations.

## Additional files

Additional file 1: Top 90% food (sub)groups that contribute to vitamin A, D and E intake, retrieved from the DNFCS (2007–2010), and most important food sources for vitamin K according to Bolton-Smith, VITADEK study, 2015–2016 (DOCX 14 kb)

Additional file 2: Schematic representation of the design of the food frequency questionnaire, VITADEK study, 2015–2016. (DOCX 1580 kb)

## Abbreviations

BNFCS2014: Belgian national food consumption survey 2014
CHCC: Child health consultation centre
DNFCS: Dutch national food consumption survey
EAR: Estimated average requirement
EFSA: European food safety authority
FFQ: Food frequency questionnaire
NPR: National population register
PANCAKE-PROJECT: Pilot study for the assessment of nutrient intake and food consumption among Kids in Europe
UI: Upper intake level
VITADEK STUDY: Study on the intake of fat-soluble vitamins A, D, E and K
WHO: World health organisation
