# Supplementary material for: Fat-soluble vitamin intake from the consumption of food, fortified food and supplements: design and methods of the Belgian VITADEK study
Source: Arch Public Health. 2017 May 16;75:31. doi: 10.1186/s13690-017-0199-3 (PMC5434571; doi:10.1186/s13690-017-0199-3)
Supplement: Supplementary file 1 — Top 90% food (sub)groups that contribute to vitamin A, D and E intake, retrieved from the DNFCS (2007–2010), and most important food sources for vitamin K according to Bolton-Smith, VITADEK study, 2015–2016 (DOCX 14 kb) [file 13690_2017_199_MOESM1_ESM.docx]

Additional file 1 90% Most contributing food (sub) groups for vitamine A, D and E retrieved from the DNFCS (2007-2010) and Most important food sources for vitamin K according to Bolton-Smith, VITADEK-study, 2015-2016

| **FOODGROUPS** | **Vitamin A** | **Vitamin D** | **Vitamin E** | **Vitamin K** |
| --- | --- | --- | --- | --- |
| Cakes and sweet biscuits | Cakes, pies, pastry | Cakes, pies, pastry | Cakes, pies, pastry |  |
| Cereals and cereal products |  |  | Breakfast cereals  Bread, crisp bread, rusks, salty biscuits, crackers | Cereals |
| Condiments and sauces | Sauces | Sauces | Sauces |  |
| Dairy products and substitutes | Cheese  Milk, milk beverages and fermented milk  Ice cream and substitutes  Cream desserts, puddings  Dairy and non dairy creams | Cheese  Cream desserts and puddings | Yoghurt  Cream desserts and pudding | Fresh cheese  Milk and milk products |
| Eggs and egg products | Eggs | Eggs | Eggs |  |
| Fat and oils | Margarines  Butter | Margarines | Margarines  Vegetable oils | Vegetable oils |
| Fruit, nuts and seeds, olives | Fruits |  | Fruits  Nuts and seeds | Fruits and nuts |
| Fish |  | Fish | Fish |  |
| Meat, meat products and substitutes | Processed meat  Poultry | Poultry  Domestic mammals/fresh meat | Processed meat  Poultry  Domestic mammals/fresh meat | Processed meat  Poultry |
| Non-alcoholic beverages | Fruit and vegetable juices |  | Fruit and vegetable juices  Carbonated soft/isotonic drinks |  |
| Sugar and confectionery | Ice cream, water ice |  | Chocolate, candy bars, paste, syrup |  |
| Vegetables | Root vegetables  Leafy vegetables  Mixed salad/vegetables  Cabbages |  | Fruiting vegetables  Cabbages  Leafy vegetables | Leafy vegetables |
