# Supplementary material for: Fat-soluble vitamin intake from the consumption of food, fortified food and supplements: design and methods of the Belgian VITADEK study
Source: Arch Public Health. 2017 May 16;75:31. doi: 10.1186/s13690-017-0199-3 (PMC5434571; doi:10.1186/s13690-017-0199-3)
Supplement: Supplementary file 2 — Schematic representation of the design of the food frequency questionnaire, VITADEK study, 2015–2016. (DOCX 1580 kb) [file 13690_2017_199_MOESM2_ESM.docx]

**ADDITIONAL FILE 2: SCHEMATIC REPRESENTATION OF THE DESIGN OF THE FOOD FREQUENCY QUESTIONNAIRE, VITADEK-STUDY, 2015-2016.**

A schematic representation of the design of the food frequency questionnaire is presented in the following print screens. Screening questions are shown in the case of breakfast cereals. First the respondent was enquired if breakfast cereals have been eaten in the past month (Question 1). If this was the case, a list of 16 brands popped up followed by a list of all marketed varieties per selected brand (question 2 and 3). Brands as well as varieties are included in the pictures. Finally consumption frequency, average portion size and amount of portions eaten per consumption day were asked for (question 4 and 5).


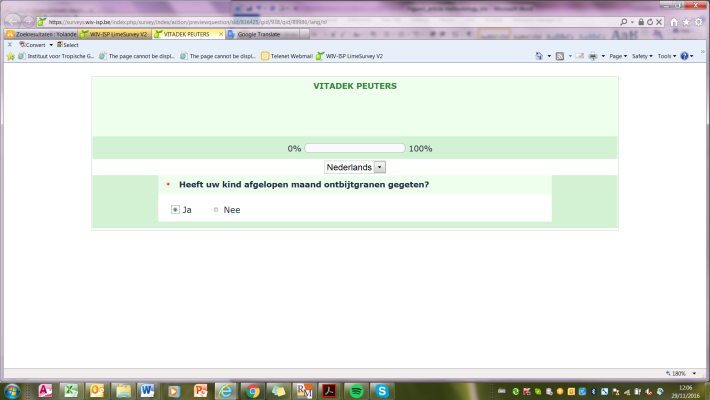


**Question 1:** Did your child eat breakfast cereals during the past month?

**Answer options:** Yes/No


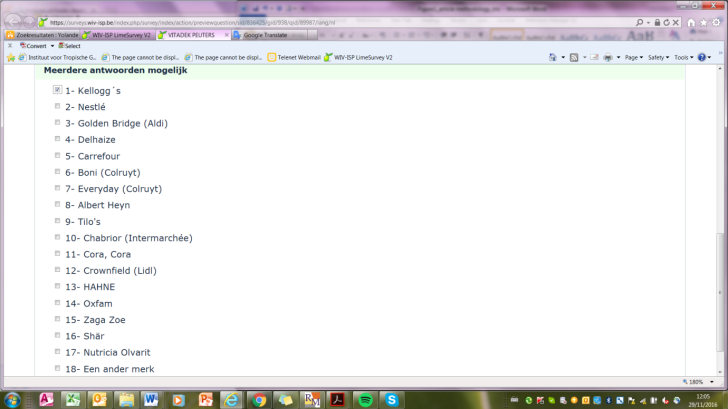

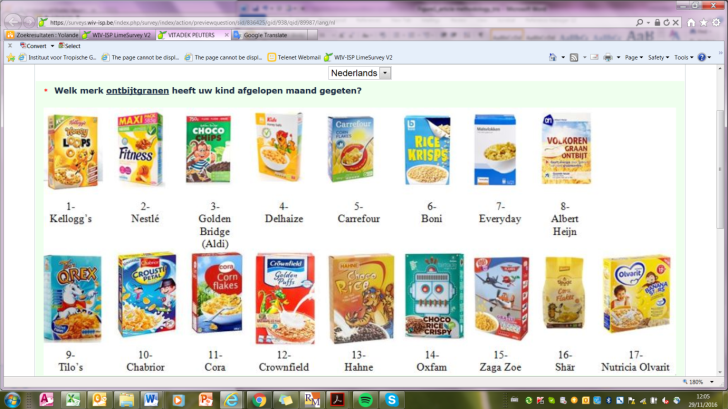


**Question 2:** Which brand(s) of breakfast cereals did your child eat during the past month?

**Answer options :** Multiple choice of brands including the option ‘other’


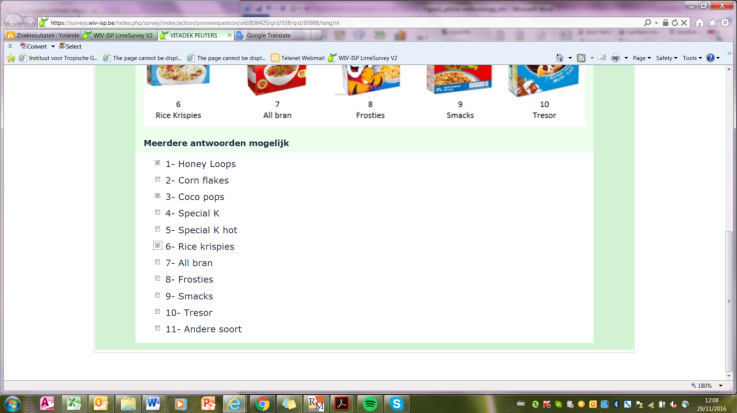

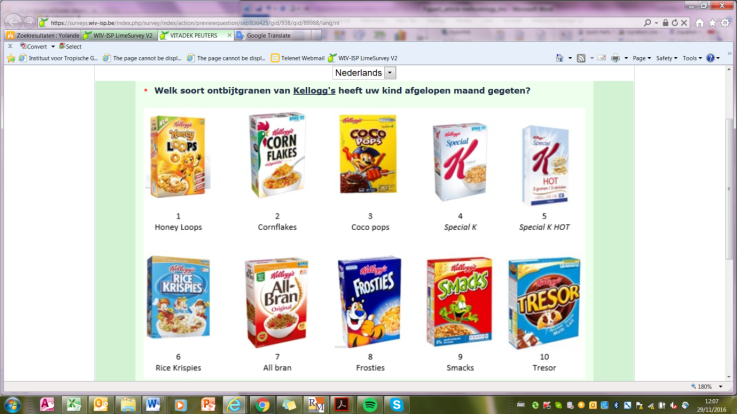


**Question 3 :** Which kind of breakfast cereals from Kellogg’s did your child eat during the past month?

**Answer options :** Multiple choice of types of Kellogg’s breakfast cereals including the option ‘other’


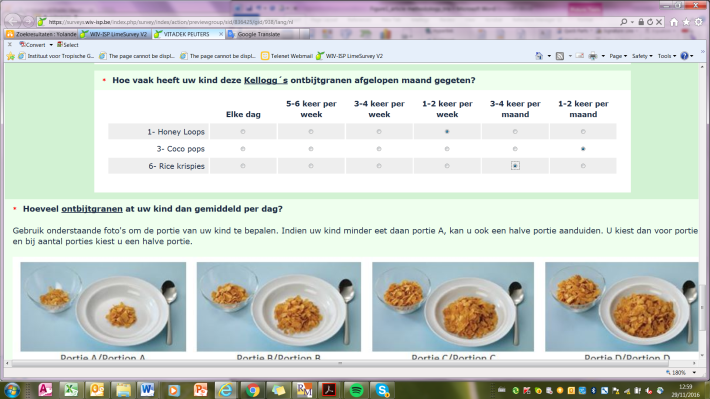


**Question 4 :** How often did your child eat these Kellogg’s breakfast cereals during the past month?

**Answer options :** Each day; 5-6 times a week; 3-4 times a week; 1-2 times a week; 3-4 times a month;

1-2 times a month


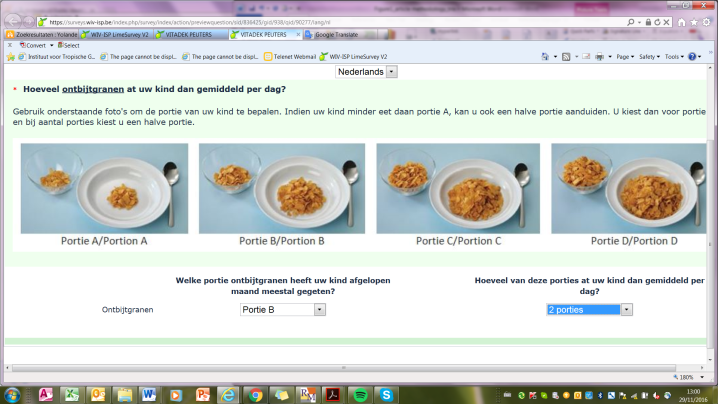


**Question 5 :** Which portion of breakfast cereals did your child then eat on average during the past month? Use the portions pictures to determine the portion your child has eaten.

- What portion did your child usually eat during the past month?

**Answer options:** portion A; portion B; portion C; portion D

- How many of these portions did your child eat on average per day?

**Answer options:** half a portion; 1 portion; 1 portion and a half; 2 portions; 3 portions; 4 portions
